# Supplementary material for: Hydrophobically gated memristive nanopores for neuromorphic applications
Source: Nat Commun. 2023 Dec 18;14:8390. doi: 10.1038/s41467-023-44019-y (PMC10728163; doi:10.1038/s41467-023-44019-y)
Supplement: Supplementary file 1 — Supplementary Information [file 41467_2023_44019_MOESM1_ESM.pdf]

Supplementary materials for Hydrophobically Gated Memristive Nanopores for Neuromorphic Applications

Gonçalo Paulo[1], Ke Sun[2,3], Giovanni Di Muccio[1], Alberto Gubbiotti[1], Jia Geng[3], Blasco Morozzo della Rocca[4], Mauro Chinappi[5], Giovanni Maglia[2], Alberto Giacomello[1] *

[1] *Dipartimento di Ingegneria Meccanica e Aerospaziale, Sapienza Università di Roma, Via Eudossiana, 18, 00184 Rome, Italy.*

[2] *Groningen Biomolecular Science & Biotechnology Institute and Center for Systems Chemistry, Stratingh Institute for Chemistry, University of Groningen, Nijenborg 4, 9747 AG, Groningen, Netherlands.*

[3] *Department of Laboratory Medicine, State Key Laboratory of Biotherapy and Cancer Center, Med+X Center for Manufacturing, West China Hospital, Sichuan University and Collaborative Innovation Center, Chengdu 610041, China.*

[4] *Department of Biology, Tor Vergata University of Rome, Via della Ricerca Scientifica 1, 00133, Rome, Italy.*

[5] *Department of Industrial Engineering, Tor Vergata University of Rome, Via del Politecnico 1, 00133, Rome, Italy.*

E-mail: alberto.giacomello@uniroma1.it

Contents:

Supplementary Note S1: Effect of transmembrane voltage on free energy. p. S-3

Supplementary Note S2: Rate calculations for the wetting and drying events. p. S-6

Supplementary Note S3: Macroscopic model based on classical nucleation. p. S-7

Supplementary Note S4: Analysing of the voltage cycles of the FraC nanopore. p. S-9

Supplementary Figure S1: Free-energy and diffusivity of pore filling. p. S-10

Supplementary Figure S2: Values of the first order coefficient, P_1, and the second order coefficient, P_2. p. S-11

Supplementary Figure S3: Estimation of the wetting/drying rates. p. S-12

Supplementary Figure S4: Collective pore wetting. p. S-13

Supplementary Figure S5: Dependence of the hysteresis area with the applied voltage. p. S-14

Supplementary Figure S6: Type of memristor behaviour. p. S-15

Supplementary Figure S7: Molecular model of FraC nanopore. p. S-16

Supplementary Figure S8: Single pore current traces at constant applied voltage and pH 3.8. p. S-17

Supplementary Figure S9: Experimental current signal of a voltage cycle of 5Hz through FraC nanopore at pH 3.8. p. S-18

Supplementary Figure S10: Experimental current signal of a voltage cycle of 2Hz through FraC nanopore at pH 3.8. p. S-19

Supplementary Figure S11: Removing the capacitive current at 5Hz and pH 3.8. p. S-20

Supplementary Figure S12: Removing the capacitive current at 2Hz and pH 3.8. p. S-21

Supplementary Figure S13: From single channel trace to average memristive behaviour. p. S-22

Supplementary Figure S14: Effect of the voltage on the free energy profile of FraC nanopore. p. S-23

Additional References: p. S-23

Supplementary Note 1.

Effect of transmembrane voltage on free energy

In this section we explore the effect that a transmembrane voltage has on the free energy profile associated with a given colective variable, which in our case is the probability of having the pore filled. Here the main assumption is that the system is at the equilibrium after applying the voltage. Consider a system described by the vector $\boldsymbol{x}$ of positions and momenta of its N particles $\boldsymbol{x} = (\boldsymbol{r_1}, \boldsymbol{p_1}, \ldots, \boldsymbol{r_N}, \boldsymbol{p_N})$, with an Hamiltonian made by a "zero voltage" part H_0 and the contribution of the external voltage V

$$H(\boldsymbol{x}, V) = H_0(\boldsymbol{x}) - Q_z(\boldsymbol{x})V \ , \tag{S1}$$

with Q displacement charge

$$Q(\boldsymbol{x}) = \frac{\pi(\boldsymbol{x})}{L} \ , \tag{S2}$$

where

$$\pi(\boldsymbol{x}) = \sum_{i=1}^{N} q_i \boldsymbol{r}_i \cdot \hat{\boldsymbol{n}} \tag{S3}$$

is the total dipole moment of the system in the direction in which the voltage is applied $\hat{\boldsymbol{n}}$, and L is the extension of the system in the same direction.

The probability density function of the system in the canonical ensemble is then

$$\rho(\boldsymbol{x}, V) = \frac{1}{Z(V)} e^{-\beta H(\boldsymbol{x}, V)} \ , \tag{S4}$$

with Z partition function

$$Z(V) = \int e^{-\beta H(\boldsymbol{x}, V)} \mathrm{d}\boldsymbol{x} \ . \tag{S5}$$

Choosing a collective variable θ which gives a coarser description of the system, the probability density of that variable is

$$P(\theta, V) = \int \delta\left(\theta - \hat{\theta}(\boldsymbol{x})\right) \rho(\boldsymbol{x}, V) \mathrm{d}\boldsymbol{x} = \frac{Z_\theta(\theta, V)}{Z(V)} \ , \tag{S6}$$

where $\hat{\theta}(\boldsymbol{x})$ is the function which associates the microscopic state to a value of the collective variable, and Z_θ is the partition function restrained to a given value of the collective variable

$$Z_\theta(\theta, V) = \int \delta\left(\theta - \hat{\theta}(\boldsymbol{x})\right) e^{-\beta H(\boldsymbol{x}, V)} \mathrm{d}\boldsymbol{x} \ . \tag{S7}$$

The associated free energy is

$$F(\theta, V) = -\beta \log P(\theta, V) \ , \tag{S8}$$

and its variation with respect to voltage

$$\frac{\partial F(\theta, V)}{\partial V} = -\frac{\beta}{P(\theta, V)} \frac{\partial P(\theta, V)}{\partial V} \ . \tag{S9}$$

The derivative of the coarse grained probability with respect to voltage is

$$\frac{\partial P(\theta, V)}{\partial V} = \int \delta\left(\theta - \hat{\theta}(\boldsymbol{x})\right) \frac{\partial \rho(\boldsymbol{x}, V)}{\partial V} \mathrm{d}\boldsymbol{x} \ , \tag{S10}$$

with

$$\frac{\partial \rho(\boldsymbol{x}, V)}{\partial V} = \left(\frac{\beta Q(\boldsymbol{x})}{Z(V)} - \frac{\mathrm{d}Z}{\mathrm{d}V} \frac{1}{Z(V)^2}\right) e^{-\beta H(\boldsymbol{x}, V)} \tag{S11}$$

and

$$\frac{\mathrm{d}Z(V)}{\mathrm{d}V} = \int \beta Q(\boldsymbol{x}) e^{-\beta H(\boldsymbol{x}, V)} \mathrm{d}\boldsymbol{x} = \beta Z(V) \left\langle Q \right\rangle_V \ . \tag{S12}$$

Hence, we obtain an expression for the derivative of the microscopic probability density with respect to voltage

$$\frac{\partial \rho(\boldsymbol{x}, V)}{\partial V} = \frac{\beta}{Z(V)} \left(Q(\boldsymbol{x}) - \left\langle Q \right\rangle_V\right) e^{-\beta H(\boldsymbol{x}, V)} \ , \tag{S13}$$

which can be used to compute the same for the coarse grained probability density

$$\frac{\partial P(\theta, V)}{\partial V} = \int \delta\left(\theta - \hat{\theta}(\boldsymbol{x})\right) \frac{\beta}{Z(V)} \left(Q(\boldsymbol{x}) - \left\langle Q \right\rangle_V\right) e^{-\beta H(\boldsymbol{x}, V)} \mathrm{d}\boldsymbol{x} \ , \tag{S14}$$

simplifying

$$\frac{\partial P(\theta, V)}{\partial V} = P(\theta) \left(\left\langle Q \right\rangle_{\theta, V} - \left\langle Q \right\rangle_V\right) \ . \tag{S15}$$

We finally obtain an expression for the first order derivative of the free energy with respect to voltage

$$\frac{\partial F(\theta, V)}{\partial V} = -\beta \left(\left\langle Q \right\rangle_{\theta, V} - \left\langle Q \right\rangle_V\right) \tag{S16}$$

To obtain the second order derivative

$$\frac{\partial F(\theta, V)^2}{\partial^2 V} = -\beta \left(\frac{\partial \left\langle Q \right\rangle_{\theta, V}}{\partial V} - \frac{\partial \left\langle Q \right\rangle_V}{\partial V}\right) \ , \tag{S17}$$

we need to compute the derivative of the expected averages. Consider the expected average of a generic

observable A

$$\frac{\partial \langle A \rangle_V}{\partial V} = \int A(\boldsymbol{x}) \frac{\partial \rho(\boldsymbol{x}, V)}{\partial V} \mathrm{d}\boldsymbol{x} \ , \tag{S18}$$

we can use Eq(S13)

$$\frac{\partial \langle A \rangle_V}{\partial V} = \int \frac{\beta A(\boldsymbol{x})}{Z(V)} \left(Q(\boldsymbol{x}) - \langle Q \rangle_V \right) e^{-\beta H(\boldsymbol{x}, V)} \mathrm{d}\boldsymbol{x} \ , \tag{S19}$$

and finally we get

$$\frac{\partial \langle A \rangle_V}{\partial V} = \beta \left(\langle AQ \rangle_V - \langle A \rangle_V \langle Q \rangle_V \right) \ . \tag{S20}$$

Now we can compute the second order variation of the free energy with voltage

$$\frac{\partial F(\theta, V)^2}{\partial^2 V} = -\beta^2 \left(\left\langle Q^2 \right\rangle_{\theta,V} - \langle Q \rangle_{\theta,V}^2 - \left\langle Q^2 \right\rangle_V + \langle Q \rangle_V^2 \right) \ . \tag{S21}$$

The free energy dependence on the voltage can be then approximated as:

$$F(\theta, V) = F(\theta) + P_1 V + P_2 V^2 \ , \tag{S22}$$

where $P_1 = -\beta \langle Q \rangle_\theta$ and $P_2 = -\beta^2 (\left\langle Q^2 \right\rangle_\theta - \langle Q \rangle_\theta^2)$. During the restrained molecular dynamics simulations used to compute the profiles of Supp. Fig. S1 we P_1 and P_2 for different filling levels, Supp. Fig. S2.

Because our pore is i) symmetric, ii) uncharged and iii) the displacement charge enters as a factor proportional to voltage, the displacement charge must not depend on the filling of the pore, as positive and negative voltages have the same effect on wetting and drying. Hence, we considered the displacement charge constant and independent of the filling level, i.e. we can discard the first order dependence on the applied voltage. The variance of the displacement sharply increases when the pore goes from dry state to the wet state, with the switching happening close at the same filling level where the free energy barrier occurs. We fit this behaviour with a sharp sigmoidal function. The variance of the dipole has the units of a capacitance, and the term added to the free energy could be related to the energy stored in the pore if it were a capacitor. The dry pore has the same shape of the wet pore, but it is filled with a lower dielectric (air vs water) and, so, the capacitance of the dry pore is lower than that of the wet pore. This means that long and narrow pore will feel less the effect of the electric field, with respect to shorter and wider ones. We underline that the difference in the capacitive energy is not due to the shape of the whole pore, but only of the hydrophobic region where the bubble is observed. The sole dependence of the wetting probability on the square of the voltage has been reported in works by different authors.[S1]

Supplementary Note 2.

Rate calculations for the wetting and drying events.

To compute the wetting/drying times shown in Fig. 1c of the main text we used the rate theory[S2,S3] fed with the free energy profile $F(N, V)$, where N is the pore filling and V the applied voltage, and the state dependent diffusivity $D(N)$

$$t_{rt} = \int_{N_a}^{N_b} \frac{e^{\frac{F(N,V)}{k_B T}}}{D(N)} dN \int_{N_a}^{N_b} e^{\frac{-F(N,V)}{k_B T}} \phi(N) dN, \tag{S23}$$

with t_{rt} being the mean transition time and N_a and N_b the filling levels of the initial and final states and where $\phi(N)$, the committor, is given by

$$\phi(N) = \frac{\int_{N_a}^{N} \frac{e^{\frac{F(N',V)}{k_B T}}}{D(N')} dN'}{\int_{N_a}^{N_b} \frac{e^{\frac{F(N',V)}{k_B T}}}{D(N')} dN'}, \tag{S24}$$

Using N_a as the dry state and N_b as the filled state yields the wetting time; conversely, using N_a as the wet state and N_b as the dry state yields the drying time. Throughout the work, the wetting and drying rates are always computed using Eq S23. The rates could also be computed using Langevin dynamics as done in previous works.[S4] The (overdamped) Langevin equation associated with the pore filling N and V is

$$\frac{\mathrm{d}N(V)}{\mathrm{d}t} = -\frac{D(N)}{k_B T} \frac{\mathrm{d}F(V)}{\mathrm{d}N} + \frac{\mathrm{d}D}{\mathrm{d}N} + \eta(N)\xi(t) \tag{S25}$$

where $\eta(N)$ determines the intensity of the thermal noise, being equal to $\sqrt{2D}$, $\xi(t)$ is a white noise process and $F(N, V)$ is the free energy of the system at a given voltage. We used an overdamped Langevin code[S5] to integrate Eq (S25) using the Euler-Maruyama algorithm.[S6] We can compute the first passage time distributions $\rho_w(\tau)$ and $\rho_d(\tau)$ of the wetting/drying transitions by running the simulation multiple times, initializing the system in the wet/dry state and recording the time each simulation takes to reach the other state. Both methods give very similar results, and we plot the comparison in Supp. Fig. S3.

Supplementary Note 3.

Macroscopic model based on classical nucleation.

We used a macroscopic model to be able to estimate the parameter space for the design of an hydrophobically gated memristor. As written in the main text, we define 4 conditions that are required for an experimentally feasible HyMN:

1. The pore must be preferentially dry at $\Delta V = 0$;
2. The pore must be wet before a certain voltage threshold ΔV^*;
3. The pore must dry out "quickly" at $\Delta V = 0$ to ensure a observable transition from the wet state to the dry state;
4. The pore must wet "quickly" at the voltage threshold ΔV^* to ensure a fast transition from the dry state to the wet state.

All the conditions can be evaluated if one as access to the free energy profile associated with the wetting variable. Computing the whole profile for all the parameter space that we explored is too computationally expensive and we have resorted to some approximations to be able to partially estimate the profile. The first condition is met if the free energy associated with the dry state is lower than the free energy associated with the wet state. Classical nucleation theory, enhanced with the line tension, gives an expression for what is expected to be the difference of free energies between the wet and dry state, $\Delta\Omega$:[S7]

$$\Delta\Omega = v_p \Delta p + \gamma_{lv} A_{lv} + \gamma_{lv} \cos(\theta) A_{sv} + \tau K, \tag{S26}$$

where v_p is the volume of the pore filled with vapor, ΔP is the pressure difference between liquid and vapour, γ_{lv} is the surface tension of the liquid, A_{lv} is the area of the liquid-vapor interface, θ is the liquid contact angle with the solid material, A_{sv} is the area of the solid-vapor interface, τ is the line tension and K is the triple line length. We consider that A_{lv} is twice the area of a circle, $2\pi r_p^2$, with r_p the pore radius, which is a simplification, as the area of the spherical cap of intruded liquid is slightly bigger than the area of its circular base. We consider that A_{sv} is equal to $2\pi r_p l_p$, with l_p the length of the pore, as this is the interior area of the pore surface. We consider K to be twice the perimeter of the pore mouth. The liquid surface tension is considered to be that of water, the pressure difference is considered to be atmospheric pressure and the contact angle is varied in this study. The value of the line tension, τ, is debated in the community,[S8] and in this case was a negative value of c.a -10 pN was used.

The second condition expands on this expression by considering the effect of applied voltage. Considering the results of Supplementary Note S1, we consider that a second order term on the voltage must enter on the free energy of the dry state. This term is also proportional to the difference between the capacitance of the two states, $-\Delta C V^2$, with $\Delta C = C_{wet} - C_{dry}$. The capacitance of these two states is computed as the capacitance of a planar capacitor, $\epsilon \frac{A}{l}$, where A is the area of the pore mouth, $\pi/4d^2$, d is the diameter of the pore and l is the length of the pore and ϵ is the electric permittivity. In the dry case, we use the electric constant of vacuum, $\epsilon = \epsilon_0 = 8.852 \cdot 10^{-12} F/m^{-1}$, while in the wet case a dielectric (water) is used, and $\epsilon = \epsilon_r \epsilon_0 = 80\epsilon_0$. The third and forth conditions require estimates of the respective wetting and drying barriers. The drying barrier, $\Delta\Omega_{dry}$, is estimated using the expression used to explain experimental results of extrusion in hydrophobic nanopores:[S9]

$$\Delta\Omega_{dry} = \Delta P K1(\theta) r_p^3 + \gamma_{lv} K2(\theta) + \tau K3(\theta) r_p, \tag{S27}$$

where the expressions for K1,K2,and K3 are given in detail in other work.[S10] The wetting barrier, $\Delta\Omega_{wet}$, is obtained by summing $\Delta\Omega$ and Δ_{dry}. We then use Arrhenius law to compute the respective times that take for the pores to wet, t_{wet}, or dry, t_{dry}:

$$t_{wet/dry} = t_0 e^{\frac{\Delta\Omega_{wet/dry}}{k_B T}}, \tag{S28}$$

where t_0 is a prefactor normally considered to be in the order of picoseconds.[S9,S11] We used 10 ps for our estimates.

Supplementary Note 4.

Analysing the experimental voltage cycles of the engineered FraC nanopore.

In this section, we report the analysis done on the experimental measurements of the current-voltage cycles reported on Fig. 3d. These cycles are computed by continuously measuring the current that passes through a single nanopore, when it is submitted to a sawtooth voltage signal, and averaging over multiple realizations after removing the capacitive current. For simplicity, we will discuss this process for the cycles done at frequency 5Hz and, but the same process was done for the experiments reported in Figure 4.

In Supp. Fig. S9 and in Supp. Fig. S10 we report the voltage (panel a) and current (panel b) traces of a single realization of a voltage cycling experiment. The experiment consists of a sawtooth signal, and we use only the results of the 9 cycles highlighted in red. To enhance the statistics, this protocol is repeated multiple times, and the averages done correspond to at least 30 cycles. As can be seen in panels c of Supp. Fig. S9 and Supp. Fig. S10, the capacitive current leads to current-voltage cycles that do not go through 0. Because we want to characterize the memristive behaviour of the FraC HyMN this capacitive current needs to be removed.

In Supp. Fig. S11 and in Supp. Fig. S12 we report he respective capacitive currents (panel a), which is obtained by averaging over a voltage cycling realization (9 cycles) where the pore did not open. This allows us this current as a baseline, and if we remove it from the experimental data, the current-voltage cycles go through 0, see panel b. We are then able to average the current-voltage cycles, observing a pinched hysteresis loop (panel c).

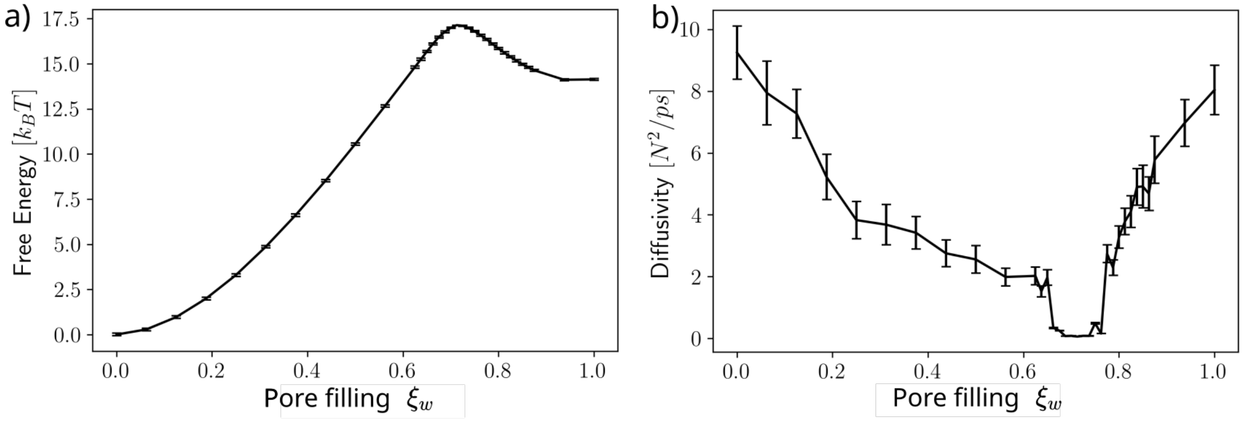

Supplementary Figure S1: **Free energy and diffusivity as a function of water filling.** We used Restrained Molecular Dynamics simulations to compute the free energy (panel a) associated with the level of water filling the model pore of Fig 1, and the corresponding state diffusivity (panel b), following the protocol described before in a previous work[S4] and in the methods section. The error on the estimation of the free energy is estimated by separating the data in 100 blocks, integrating each independently and then averaging the resulting profiles. The error on the estimation of the diffusivity is described before in a previous work.[S4] As mentioned in the main text, the pore has a stable (dry, $\xi_w = 0$) and a metastable state (wet, $\xi_w = 1$) when no voltage is applied. The diffusivity of the wetting variable depends on the value of the wetting variable, and changes more than one order of magnitude from its maximum value to its minimum value. The diffusivity of the wetting variable is necessary to compute the correct wetting and drying times and their dependence on voltage. We consider that this diffusivity does not depend on the voltage, as we have shown previously that it does not depend on the applied pressure.[S4] Here we report the water filling, which we compute as the number of water molecules inside the pore divided by the number of water molecules in the wet state. These particular results we previously reported in another work.[S4]

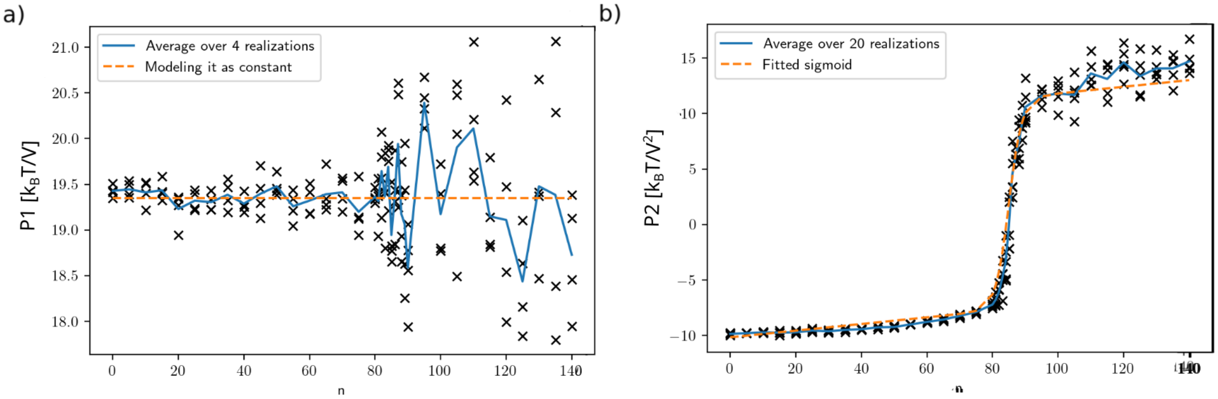

Supplementary Figure S2: **Values of the first order coefficient, P_1, and the second order coefficient, P_2.** During the restrained molecular dynamics simulations, P_1 and P_2, the first and second order coefficients on the expansion of the free energy discussed in Supp. Note 1, were calculated at different pore fillings, n. The pore filing in this figure is related to the number of water molecules inside the control box used in the RMD simulations. Because the pore is symmetric and uncharged we model P_1 as constant, panel (a), not having any effect on the free energy, as the free energy is always defined up to a constant. P_2 can be fitted by a sigmoid function plus a straight line, panel (b). Because the free energy is always defined up to a constant we set the P_2 equal to zero at the maximum of the free energy profile, n=90, corresponding to ξ_w= 0.7 in figure S1.

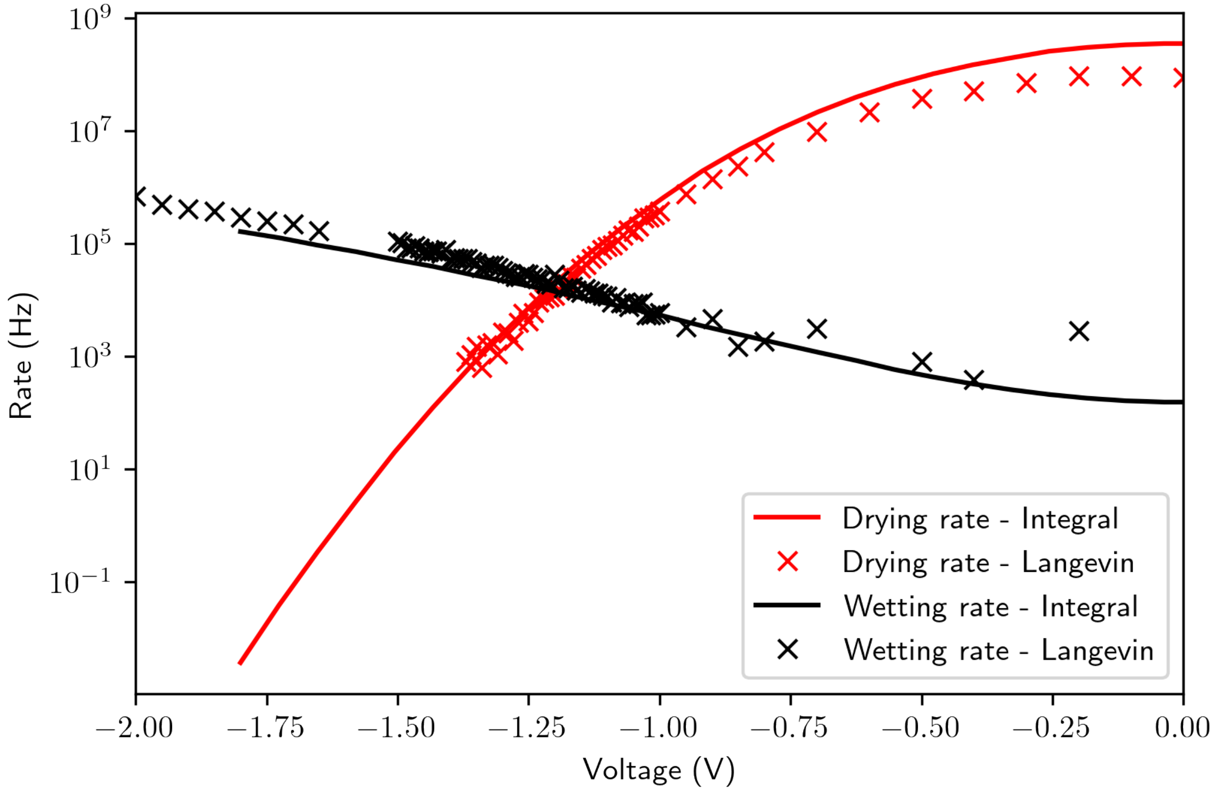

Supplementary Figure S3: **Estimation of the wetting/drying rates.** We estimate the wetting(red)/drying(black) rates using two different methods. The integral method involves solving Eq S23, and is the one use throughout this work. The other is simulating the evolution of the Langevin equation, as done in previous work.[S4] Both techniques give similar results, and due to the fact that the integral method is easier to compute, we use these results throughout the work. The point where the rates intersect is the value of V_c reported in figure 1 of the main text. The value of V_c is ca. 1.2 V.

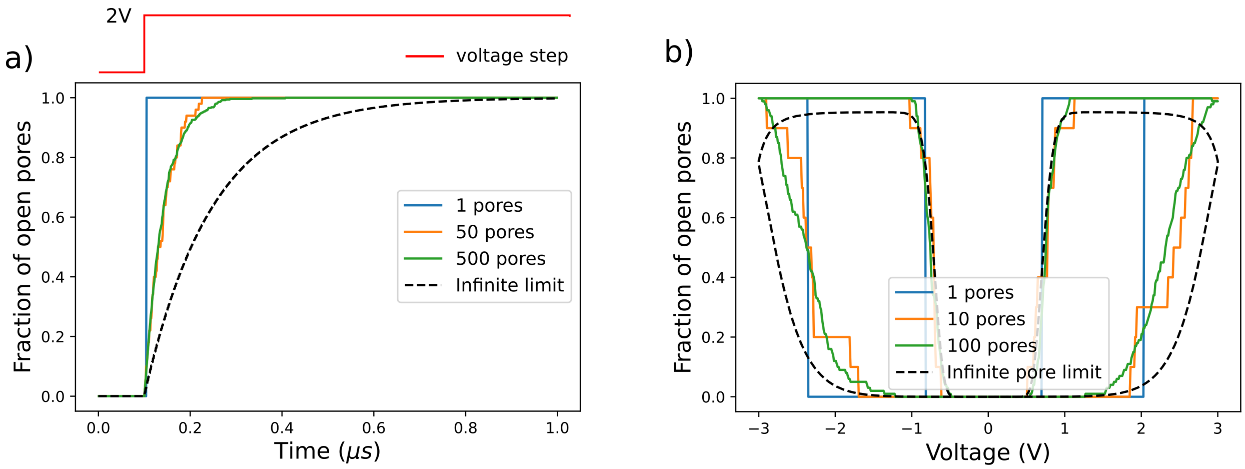

Supplementary Figure S4: **Collective pore wetting.** a) Response of different number of pores, 1, 50 and 500, when a step voltage is applied at $t_0 = 0.1$ μs, see full black line. Black dashed line is computed integrating Eq 2 of the main text, and represents the behaviour of an infinite number of pores. Colored lines are computed via a Langevin Dynamics see Supp. Fig. S3. b) Voltage cycles, computed via the same protocol as the one in Fig. 1 of the main text, for different number of pores, 1, 10 and 100. The stochastic opening of the pores is smoothed out as the number of pore increases, and just some tens of pores, between 10 and 50, are enough for a smooth behaviour. It must be noted that the limit behavior for the Langevin simulations and the infinite limit computed via Eq 2 is not the same, due to the fact that we are using the rates computed with Eq S23, and they are slightly different from the ones computed with Langevin.

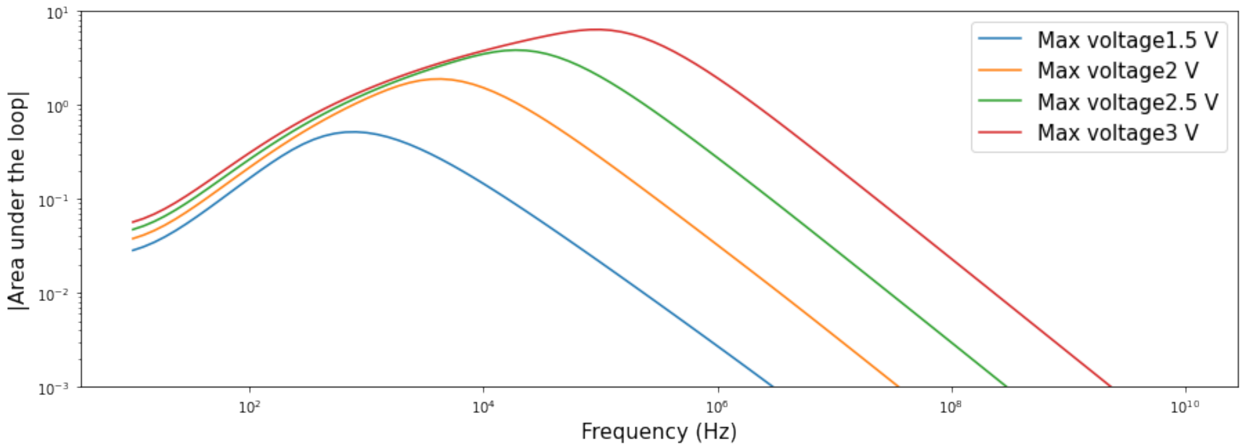

Supplementary Figure S5: **Dependence of the area under the hysteresis curve with the maximum applied voltage.** The hysteresis loop observed in Fig. 1 of the main text, and the analysis of the dependence of the area under the loop on the frequency, were done using a given maximum applied voltage (3V, corresponding to 2.5 V_c). For any given maximum applied voltage there exists a frequency that maximizes the are under the curve. In the above panel, we show how the maximizing frequency increases as the maximum applied voltage increases. The area under the curve also increases with the applied voltage. Of course even though in theory these peaks can move while increasing or decreasing the applied voltage there is a minimum current that can be measured, bounding the frequencies from below, and a maximum cycling speed that can be done, bounding the frequencies from above. Low maximum applied voltage, below V_c, would create almost indistinguishable hysteresis, and high maximum applied voltage would require very high cycling frequencies for hysteresis to be observed.

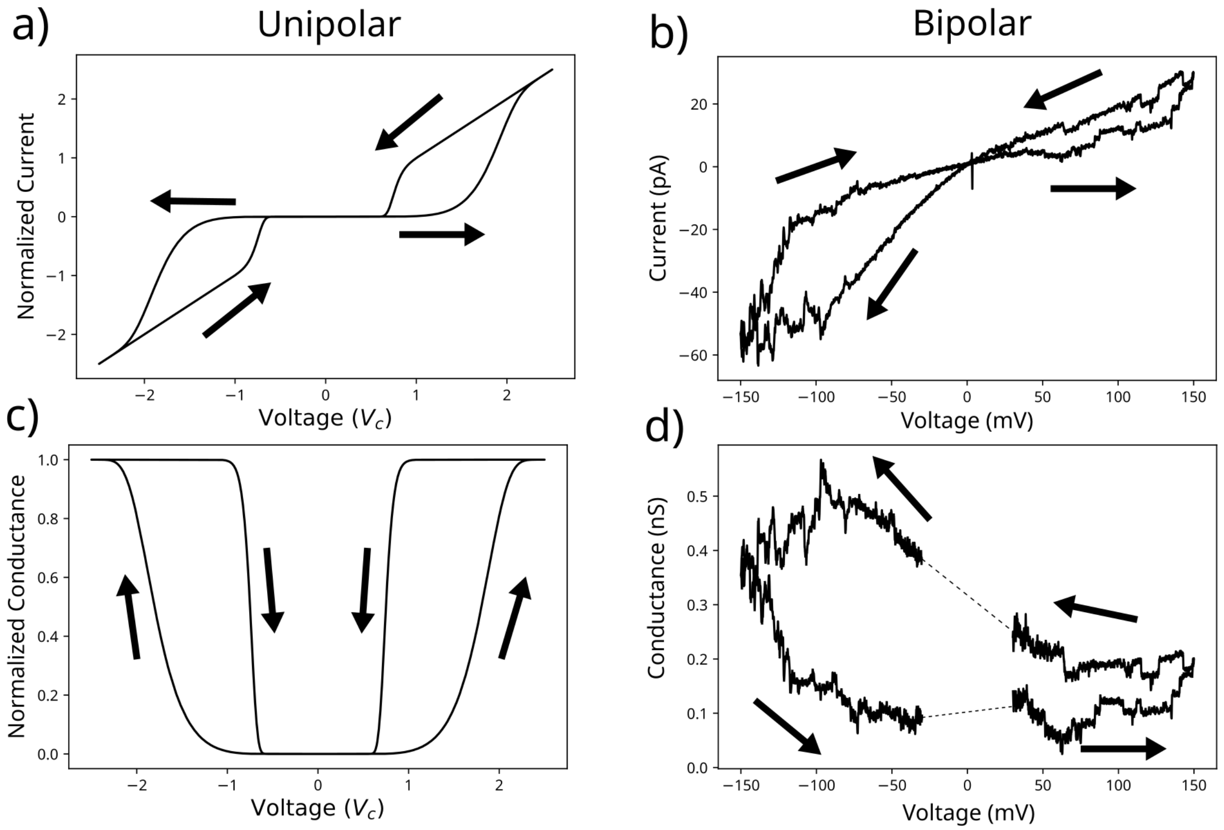

Supplementary Figure S6: **Type of memristor behaviour.** In this figure, we discuss the different type of memristor behaviour observed on hydrophobically gated memristive nanopores (HyMNs). In a) we plot the I-V curve represented in Fig.1d) of the main text, indicating the direction of the voltage cycle. The current does not self intersect at the origin, but the conductance of the system does, see panel c). This is the characteristic behaviour of an unipolar memristor. In panel b) we plot the experimental I-V curve represented in Fig.3d) of the main text, indicating the direction of the voltage cycle. The current self intersects at the origin, but the conductance of the system does not, see panel d). This is the characteristic behaviour of a bipolar memristor.

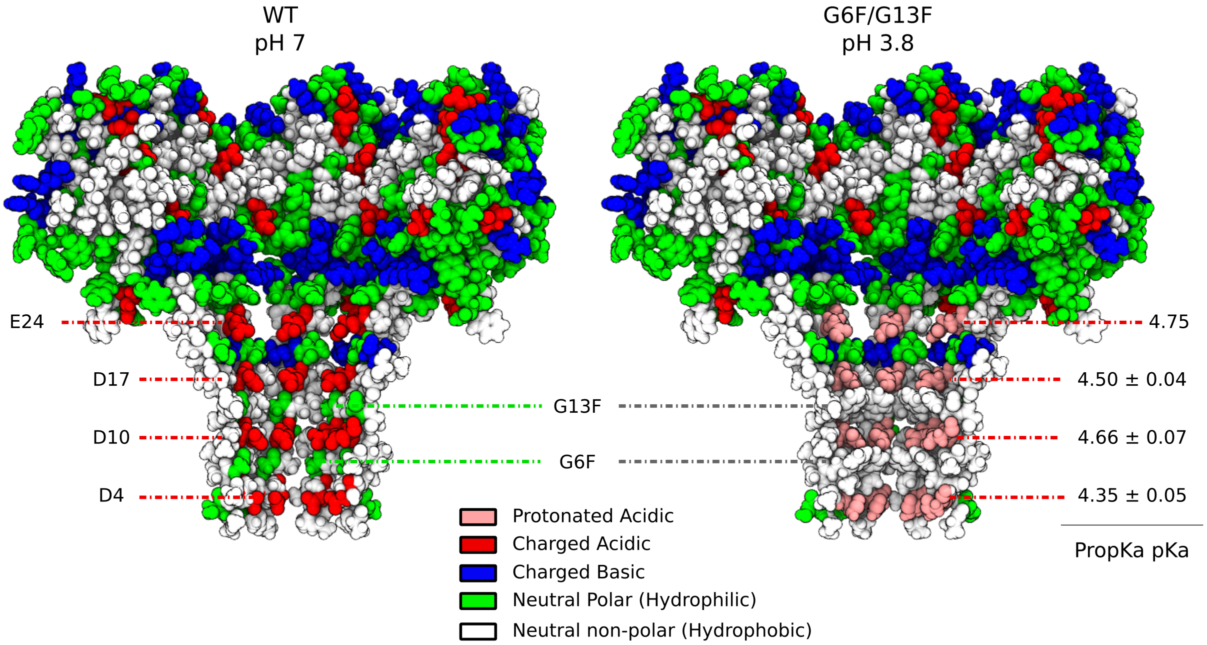

Supplementary Figure S7: **Molecular model** of WT (left) and hydrophobically engineered (right) FraC nanopore. In our experiments, the glycines G6 and G13 are mutated into high hydrophobic phenilalanines (contact angle $> 110°$).[S12] At lower pH, all the acidic residues in the constriction are completely neutralized; in this condition, the extreme confinement together with the hydrophobicity of phenilalanine residues allows the nucleation of a stable vapor bubble inside the pore constriction, see Fig. 3 of the main manuscript.

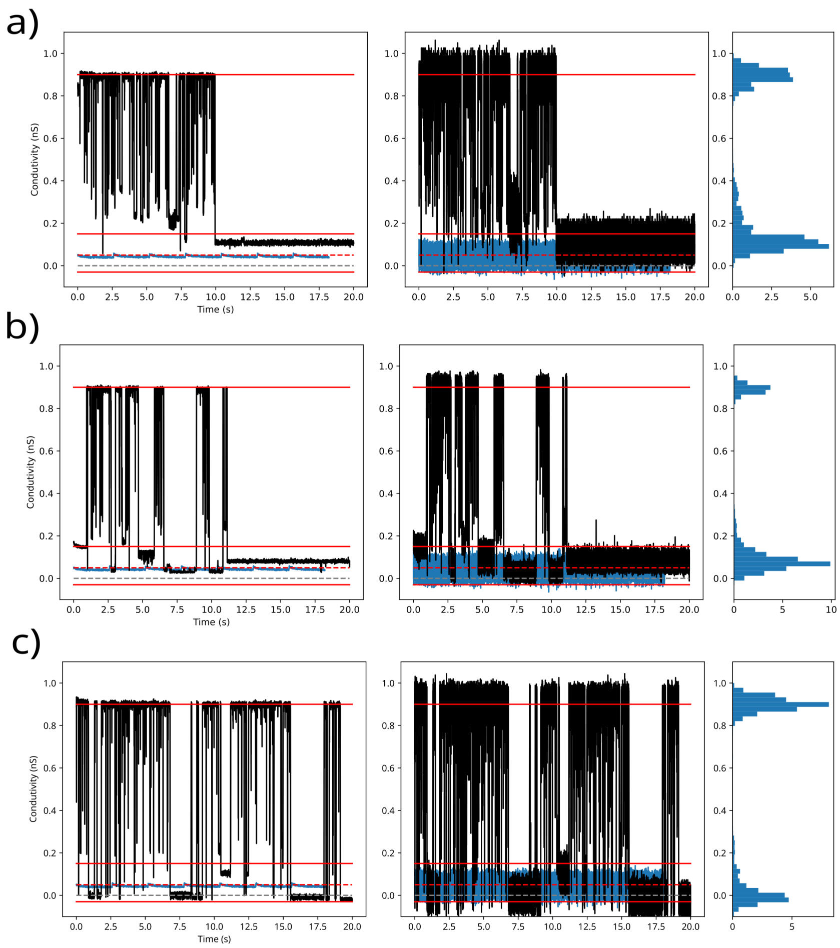

Supplementary Figure S8: **Single pore current traces at constant applied voltage and pH 3.8.** Pannels a, b and c correspond to traces at constant applied voltage, -25 mV, -50 mV and 50 mV respectivelly. In these pannels, the first graph corresponds to the recorded signal convoluted with a constant signal, in such a way as to do a moving average with a window of 0.01s. The second graph correspond to the raw signal measured. Red lines were added at 0.9 nS, the open pore conductance, and 0.15 nS the initial state of the pore at -50 mV, and at -0.05 ns the bottom conductance state at 50 mV and are the same in all the figures. The blue signal corresponds to the signal of a membrane with no pores, and the red dashed line corresponds to its average value. The grey dashed line corresponds to 0. For each of the raw signal measurement we have included an histogram for the measured conductance, which shows two clear peaks for a low conductance and an high conductance state. The data present in panel c is the same as in figure 3 c of the main text.

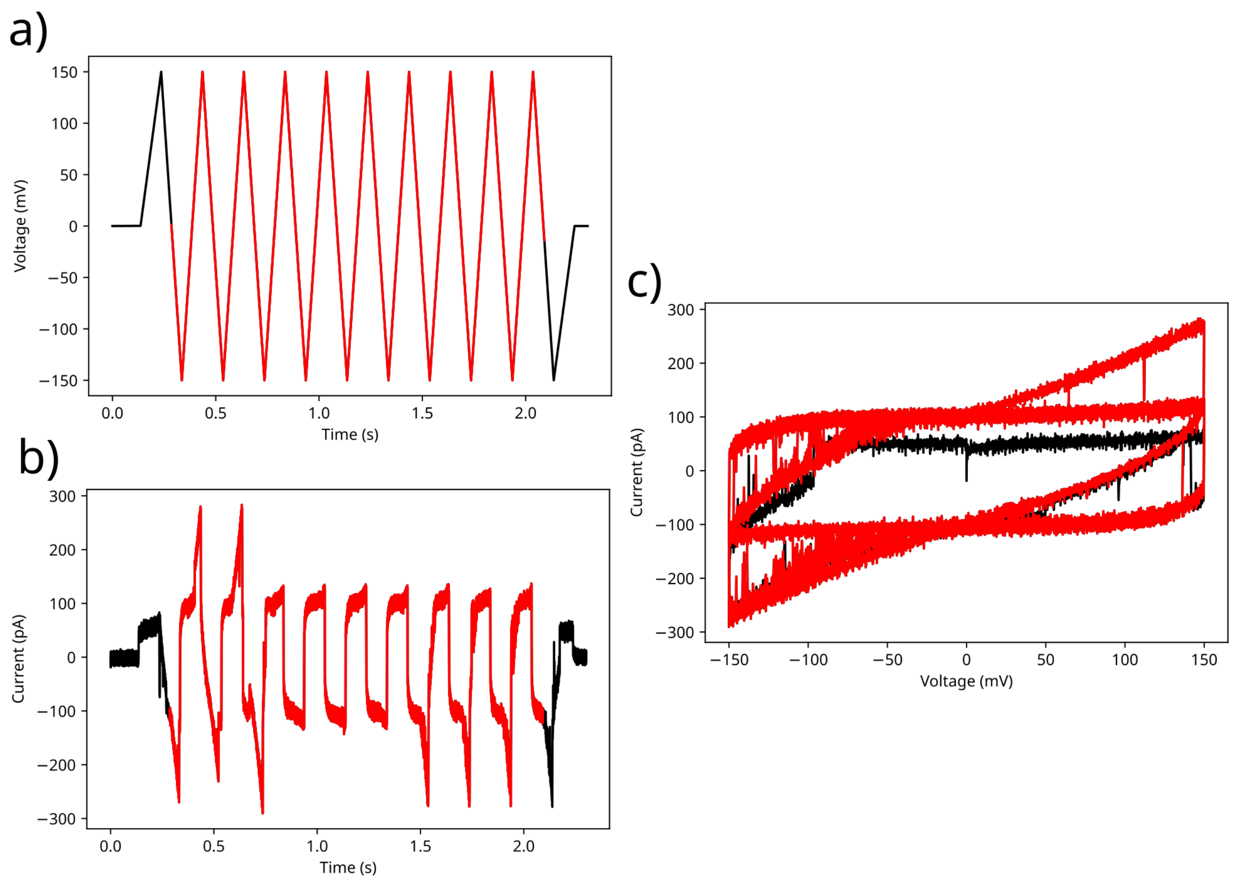

Supplementary Figure S9: **Experimental current signal of a voltage cycle of 5Hz through FraC nanopore at pH 3.8.** Panel a) corresponds to the imposed voltage trace at 5Hz, where the voltage is changed from 150 mV to -150mV and back. Panel b) corresponds to the current measured during a single realization of this experiment. The characteristic signal of the capacitive current can be observed, while the signal corresponding to the opening of the pore gives rise to "spikes" on top of that current. Panel c) shows the current-voltage curve, which does not cross zero, with a non-pinched hysteresis curved due to the capacitive current. In all panels, in red are highlighted the 9 cycles used for all the averages. In this figure, we show a single realization of the data used in figure 3d.

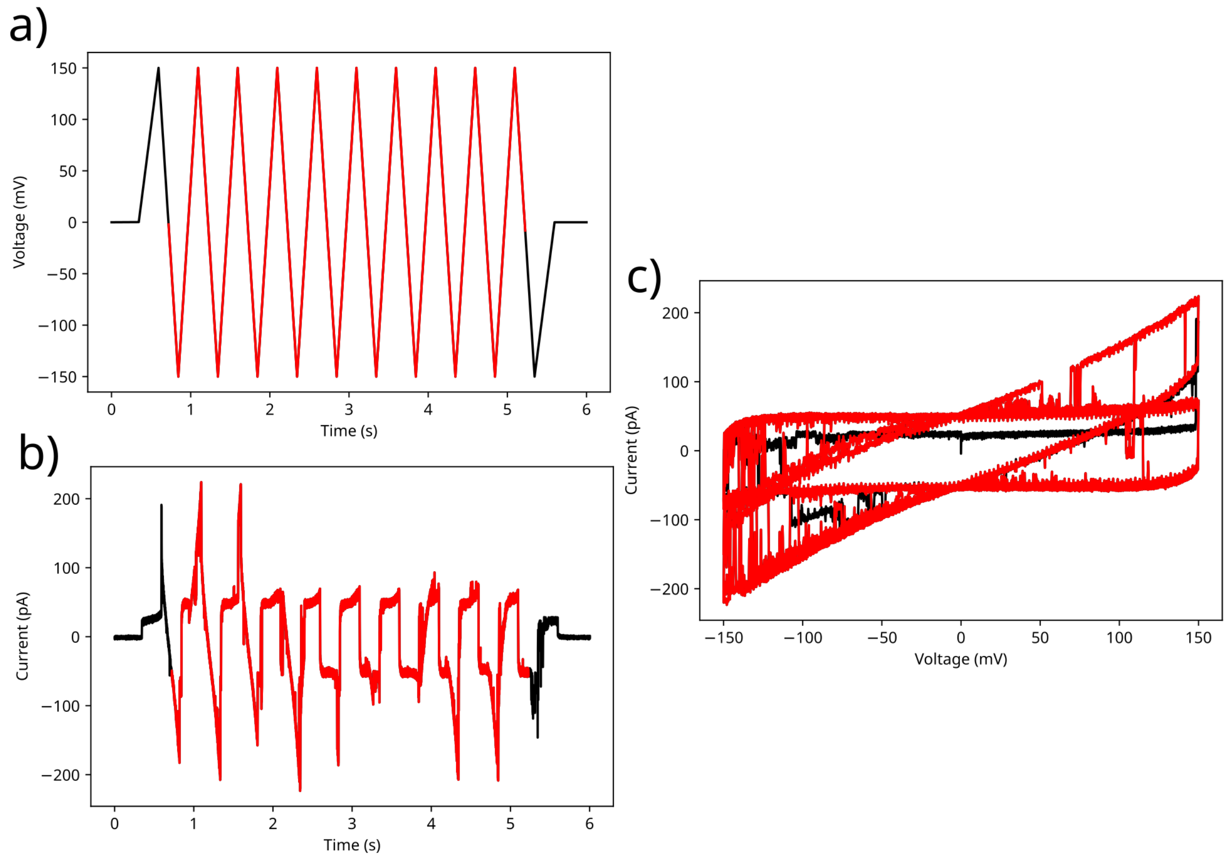

Supplementary Figure S10: **Experimental current signal of a voltage cycle of 2Hz through FraC nanopore at pH 3.8.** Panel a) corresponds to the imposed voltage trace at 2Hz, where the voltage is changed from 150 mV to -150mV and back. Panel b) corresponds to the current measured during a single realization of this experiment. The characteristic signal of the capacitive current can be observed, while the signal corresponding to the opening of the pore gives rise to "spikes" on top of that current. Panel c) shows the current-voltage curve, which does not cross zero, with a non-pinched hysteresis curved due to the capacitive current. In all panels, in red are highlighted the 9 cycles used for all the averages. In this figure, we show a single realization of the data used in figure 3d.

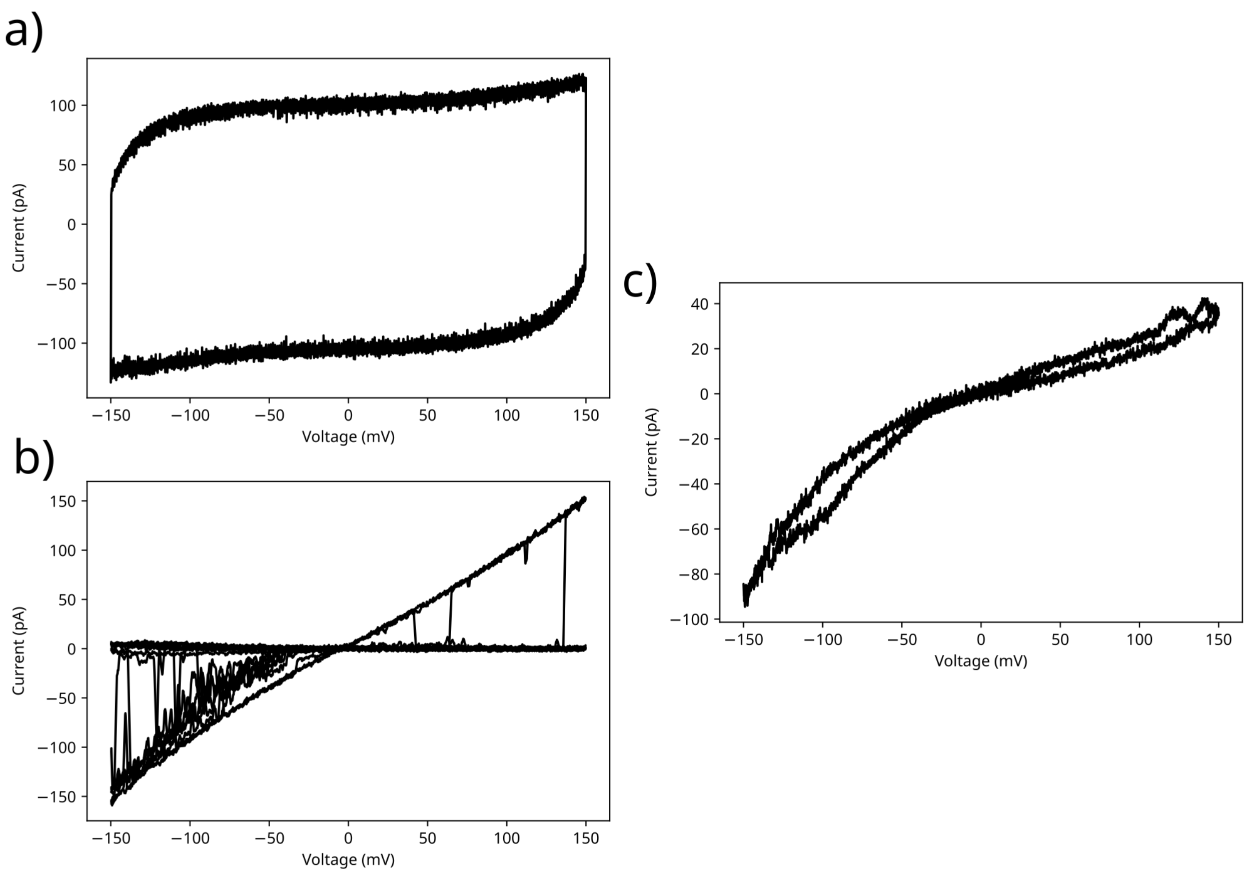

Supplementary Figure S11: **Removing the capacitive current at 5Hz and pH 3.8.** Panel a) corresponds to the average current over 9 voltage cycles where the pore never opened. The measured current is used as the baseline to remove from the other realizations. Panel b) corresponds to the subtraction of the measured current, shown in red in figure S9, and the capacitive current, with the current signal now going through 0. Panel c) shows the average current-voltage curve of a single realization, by averaging out ca. 100 voltage cycles where the pore opened at least one. This figure corresponds to the same data as figure S9 and figure 3d of the main text.

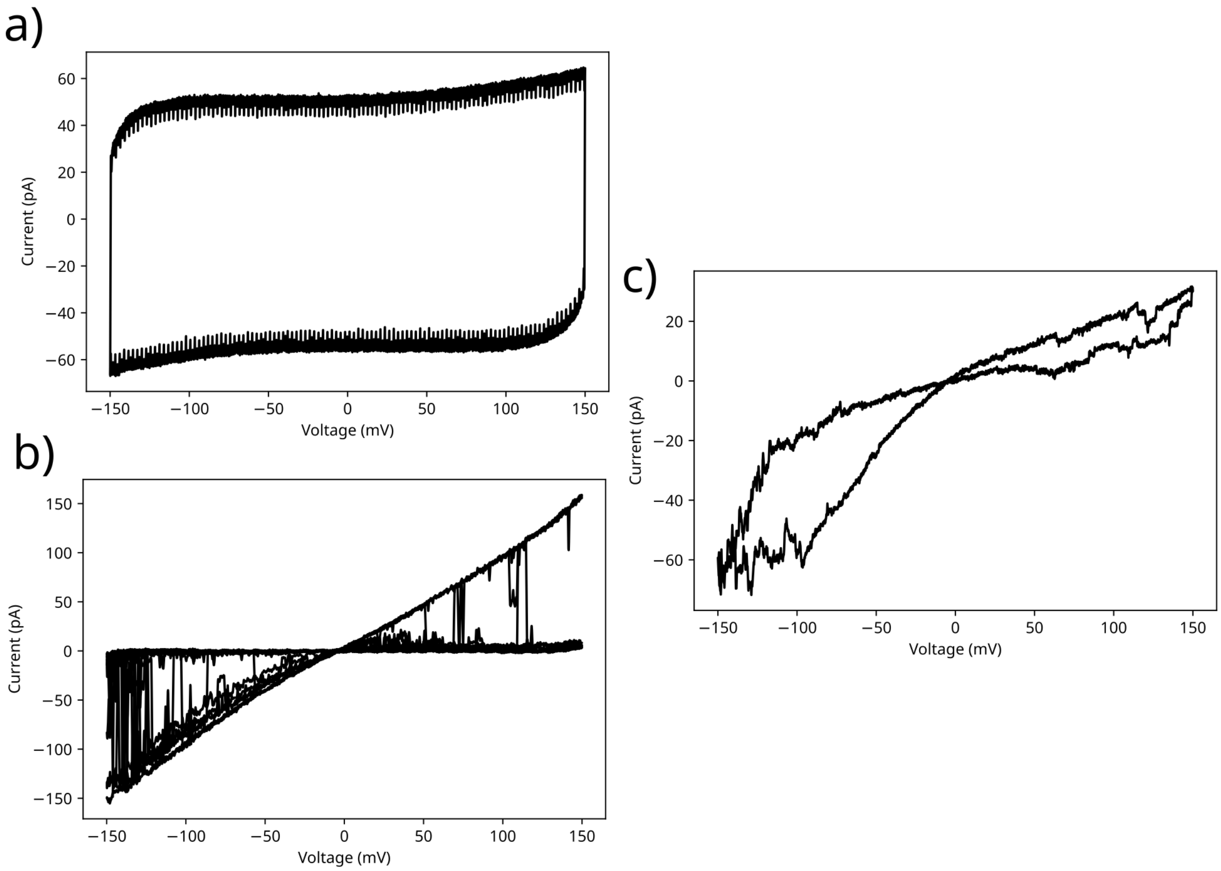

Supplementary Figure S12: **Removing the capacitive current at 2Hz and pH 3.8.** Panel a) corresponds to the average current over 9 voltage cycles where the pore never opened. The measured current is used as the baseline to remove from the other realizations. Panel b) corresponds to the subtraction of the measured current, shown in red in figure S10, and the capacitive current, with the current signal now going through 0. Panel c) shows the average current-voltage curve of a single realization, by averaging out ca. 40 voltage cycles where the pore opened at least one. This figure corresponds to the same data as figure S10 and figure 3d of the main text.

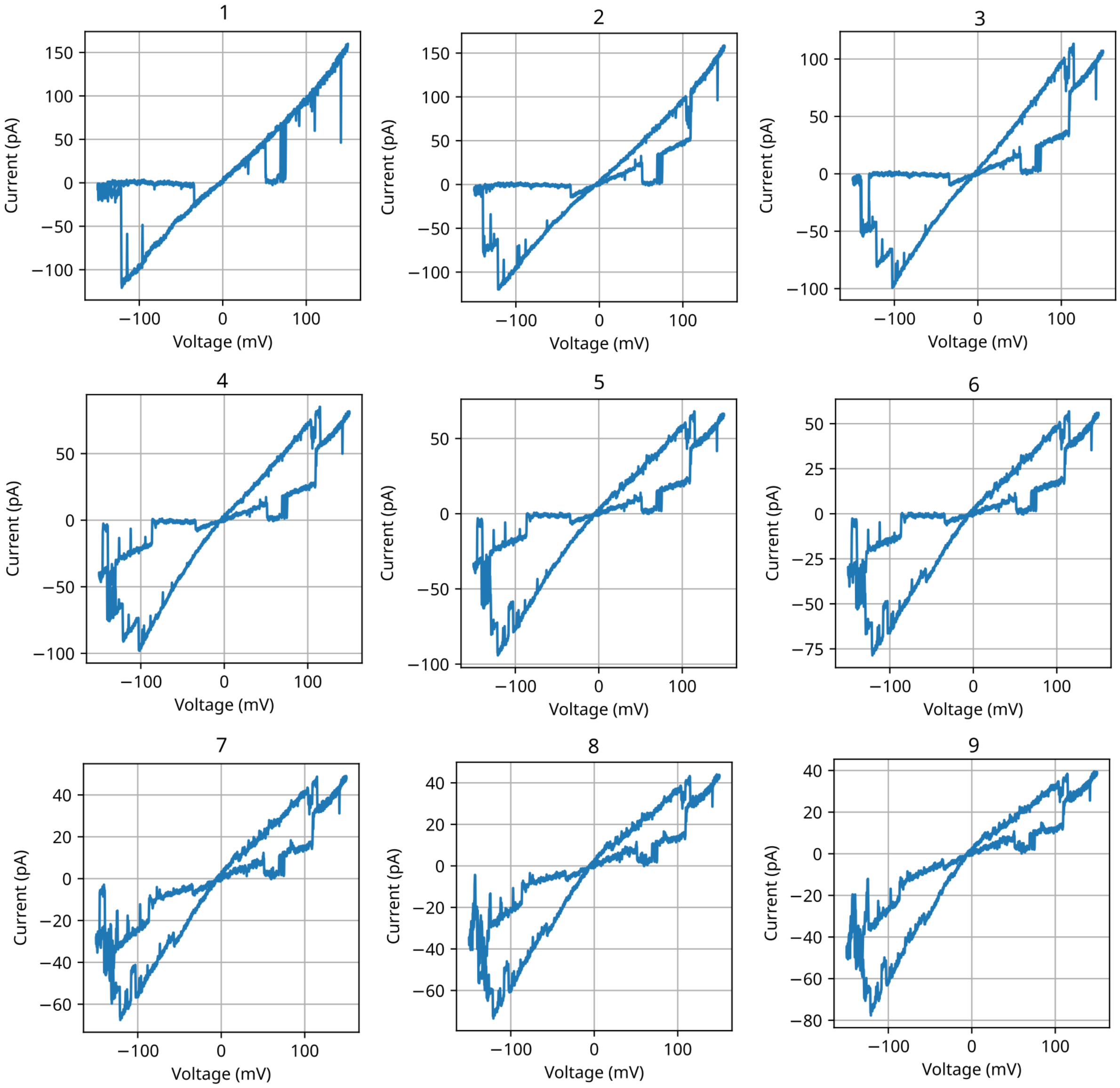

Supplementary Figure S13: **From single channel trace to average memristive behaviour at 2Hz and pH 3.8.** Panel 1 corresponds to a single voltage cycle measurement of a single pore at 2Hz and at pH 3.8. Panels 2 to 9 correspond to averaging the signal for that number of consecutive cycles, e.g. panel 9 corresponds to the averaging of 9 consecutive cycles. Single channel recordings have the trace of stochastic memristors, and averaging the cycles makes the traditional pinched loop stand out. This data corresponds to the same data shown in figures S10, S12 and figure 3d of the main text.

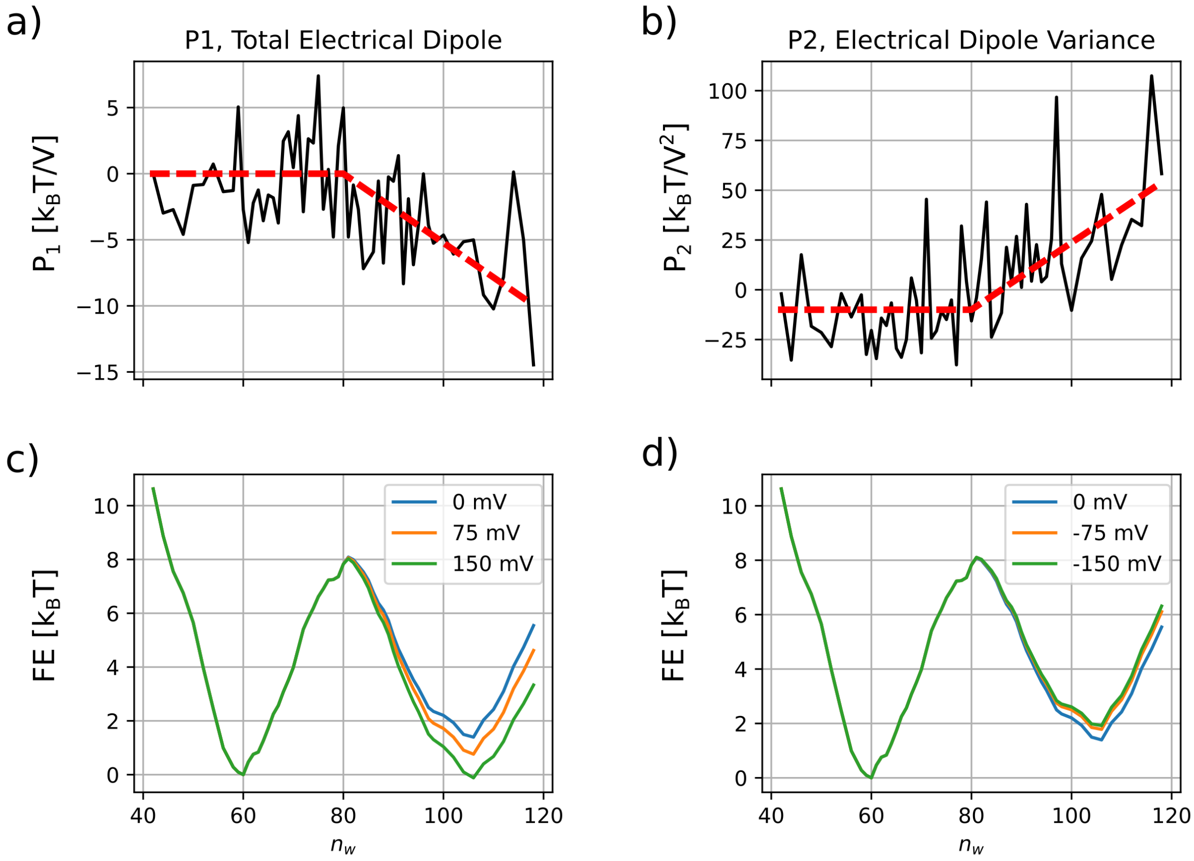

Supplementary Figure S14: **Effect of the voltage on the free energy profile of FraC nanopore.** Values of the first order coefficient, P1 (panel a), and the second order coefficient, P2 (panel b) on the expansion of the free energy discussed in Supp. Note S1, Eq.S25, obtained from the RMD simulations of the FraC system shown in Fig. 3 of the main manuscript. The P1 and P2 value used in the expansion are the averaged profiles shown by the red dashed lines. Panel c) show the free energy profile changes at positive applied voltages, while panel d) shows it at negative voltages. It can be noted the asymmetric tilting of the free energy profile under opposite voltages: at positive voltages, the model predict that the wet and dry states becomes equiprobable at around 150 mV, while keeping the wetting barrier the same. Differently, at negative voltages the dry state remains the most probable up to -150 mV; moreover, it is interesting to note that our theory predicts, at negative voltages, that the electric field reduces the drying barrier, in opposition to what is commonly expected in electrowetting. This is due to the interplay of intrinsic dipoles present into the system and the external electric field. However, a comprensive analysis of this "electrodrying" phenomena will require a dedicated study.

Supplementary References

[S1] Trick, J. L.; Song, C.; Wallace, E. J.; Sansom, M. S. P. Voltage Gating of a Biomimetic Nanopore: Electrowetting of a Hydrophobic Barrier. *ACS Nano* **2017**, *11*, 1840–1847.

[S2] Hänggi, P.; Talkner, P.; Borkovec, M. Reaction-rate theory: fifty years after Kramers. *Reviews of Modern Physics* **1990**, *62*, 251–341.

[S3] Zhu, F.; Hummer, G. Theory and simulation of ion conduction in the pentameric GLIC channel. *Journal of chemical theory and computation* **2012**, *8*, 3759–3768.

[S4] Paulo, G.; Gubbiotti, A.; Giacomello, A. An atomistically informed multiscale approach to the intrusion and extrusion of water in hydrophobic nanopores. *The Journal of Chemical Physics* **2023**, *158*.

[S5] Gubbiotti, A.; Chinappi, M.; Casciola, C. M. Confinement effects on the dynamics of a rigid particle in a nanochannel. *Physical Review E* **2019**, *100*, 053307.

[S6] Miguel, M. S.; Toral, R. *Instabilities and nonequilibrium structures VI*; Springer, 2000; pp 35–127.

[S7] Giacomello, A.; Roth, R. Bubble formation in nanopores: a matter of hydrophobicity, geometry, and size. *Advances in Physics: X* **2020**, *5*, 1817780.

[S8] Tinti, A.; Giacomello, A.; Meloni, S.; Casciola, C. M. Classical nucleation of vapor between hydrophobic plates. *The Journal of Chemical Physics* **2023**, *158*, 134708.

[S9] Lefevre, B.; Saugey, A.; Barrat, J.-L.; Bocquet, L.; Charlaix, E.; Gobin, P.-F.; Vigier, G. Intrusion and extrusion of water in hydrophobic mesopores. *The Journal of chemical physics* **2004**, *120*, 4927–4938.

[S10] Guillemot, L.; Biben, T.; Galarneau, A.; Vigier, G.; Charlaix, É. Activated drying in hydrophobic nanopores and the line tension of water. *Proceedings of the National Academy of Sciences* **2012**, *109*, 19557–19562.

[S11] Tinti, A.; Giacomello, A.; Grosu, Y.; Casciola, C. M. Intrusion and extrusion of water in hydrophobic nanopores. *Proceedings of the National Academy of Sciences* **2017**, *114*, E10266–E10273.

[S12] Zhu, C.; Gao, Y.; Li, H.; Meng, S.; Li, L.; Francisco, J. S.; Zeng, X. C. Characterizing hydrophobicity of amino acid side chains in a protein environment via measuring contact angle of a water nanodroplet on planar peptide network. *Proceedings of the National Academy of Sciences* **2016**, *113*, 12946–12951.
